# An unusual cause of inappropriate shocks delivered by an implantable cardioverter defibrillator

**DOI:** 10.1186/s12872-024-04038-z

**Published:** 2024-07-23

**Authors:** Benito Baldauf, Ernest W. Lau, Marzia Giaccardi, Hendrik Bonnemeier

**Affiliations:** 1 Christian-Albrechts-Universität, Medizinische Fakultät, Christian-Albrechts-Platz 4, 24118 Kiel, Germany; 2 Hochschule Bremerhaven, Life Sciences, An d. Karlstadt 8, 27568 Bremerhaven, Germany; 3 Royal Victoria Hospital, 274 Grosvenor Road, Belfast, BT12 6BA UK; 4 Osp. Santa Maria Annunziata, Via Antella 58, Bagno a Ripoli, 50012 Italy

**Keywords:** Cardiac implantable electronic device, Severe adverse event, Twiddler’s syndrome, Lead extraction, Taurolidine

## Abstract

**Introduction:**

Cardiac implantable electronic device (CIED) complications present significant challenges in clinical practice, especially in elderly patients with multiple comorbidities. Common adverse events include infection, lead malfunction, and device migration. Twiddler’s Syndrome, a rare but serious CIED complication characterised by patient manipulation causing lead displacement and device malfunction, is often underreported. The literature consists mainly of case reports and small series, providing limited guidance on prevention and management. As CIEDs are critical for managing cardiac arrhythmias and heart failure, understanding and addressing Twiddler’s Syndrome is essential. This case report aims to contribute to the literature by detailing a case of Twiddler’s Syndrome, emphasising the importance of a multidisciplinary approach for optimal management.

**Case Presentation:**

A 59-year-old male presented with discomfort around his implantable cardioverter defibrillator (ICD) site and the sternal area over the past two days. He denied pain, dyspnoea, or dizziness. Clinical examination revealed a normal heart rhythm and no peripheral pulse deficit. Ultrasound revealed a reduced left ventricular ejection fraction. The atrial lead was not visible, and the shock coil was misplaced. ICD interrogation showed inappropriate shocks due to sensing artifacts and exit block in both leads, with no arrhythmias detected. An X-ray confirmed lead dislodgement and significant entanglement in the pocket. The patient was diagnosed with Twiddler’s Syndrome and scheduled for surgical revision.

**Discussion/Conclusions:**

Dilated cardiomyopathy (DCM), characterised by left ventricular dilatation and dysfunction, accounts for a significant proportion of systolic heart failure cases. Despite advancements in heart failure management, DCM patients remain at high risk for sudden cardiac death (SCD), making ICD implantation crucial. However, CIED placement carries risks of complications, including Twiddler’s Syndrome. This condition can lead to lead dislodgement and device malfunction, resulting in inappropriate shocks and potential patient harm. In this case, a single-session extraction and re-implantation were successfully performed using a multidisciplinary approach, emphasising the importance of comprehensive management strategies to address such complications effectively. Regular follow-up showed no adverse events, highlighting the procedure’s success and the potential benefits of using advanced antimicrobial adjuncts to prevent infections. This case underscores the need for awareness and standardised protocols for managing Twiddler’s Syndrome to improve patient outcomes in the growing population of CIED recipients.

## Introduction

Cardiac implantable electronic device (CIED) complications, in general, pose a substantial burden on the healthcare system, with infection, lead malfunction, and device migration being among the most common adverse events. Despite the advancements in CIED technology and implantation techniques, the management of these complications remains challenging, particularly in elderly patients with multiple comorbidities.

Twiddler’s Syndrome represents a unique subset of CIED complications that is often underreported and may be overlooked in clinical practice. The literature on Twiddler’s Syndrome is limited to case reports and small case series, providing insufficient guidance for clinicians regarding optimal prevention and management strategies. Moreover, current clinical guidelines primarily focus on more common complications, leaving a gap in standardised protocols for addressing Twiddler’s Syndrome specifically.

Given the critical role of CIEDs in managing cardiac arrhythmias and heart failure, a deeper understanding of Twiddler’s Syndrome and the development of comprehensive management guidelines are essential. This case report aims to contribute to the existing literature by detailing the presentation, diagnosis, and surgical management of Twiddler’s Syndrome, highlighting the importance of a multidisciplinary approach involving a heart team to optimise patient outcomes.

## Case presentation

A 59-year-old male patient presented to our outpatient clinic. He reported of discomfort in the area of his implantable cardioverter defibrillator (ICD) generator and the sternal area during the last two days.

The patient did not report any pain, dyspnoea or dizziness. During inspection and auscultation, the patient presented in a compensated state with normal regular heart rhythm and no peripheral pulse deficit. The inspection of the implantation site of the ICD (left clavicular mid caudal subpectoral position) was uneventful. An electrocardiogram (ECG) revealed normal sinus rhythm without excitation propagation and regression disorders.

**Fig. 1:**
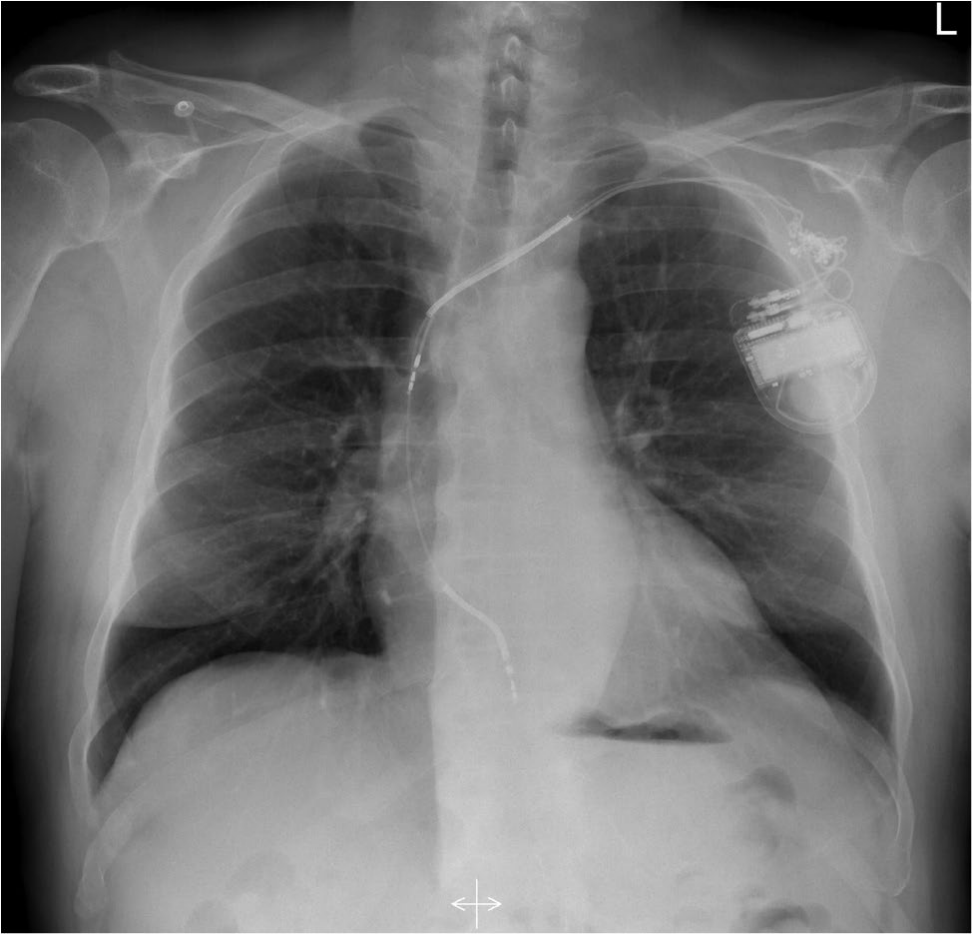
X-ray: dislodgement of the ventricular lead (projected to the area of the tricuspid annulus) and the atrial lead (tip projected to the superior vena cava)

**Fig. 2:**
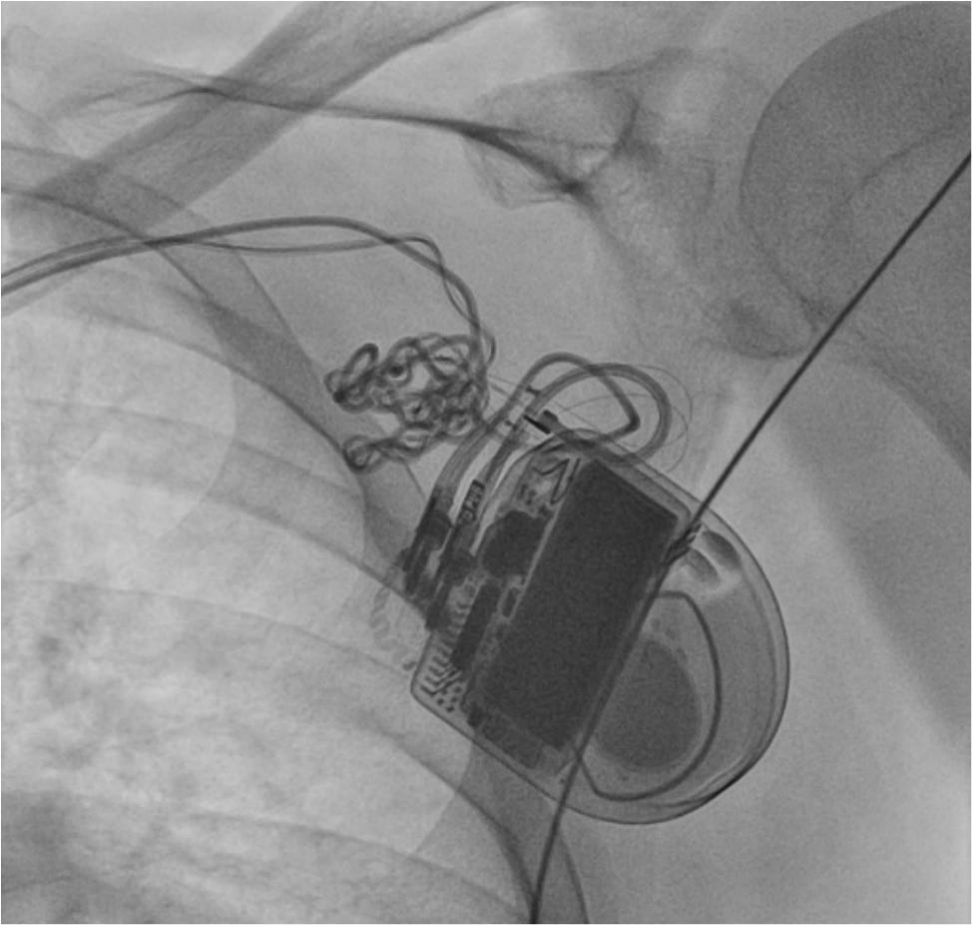
Fluoroscopic image zooming in on the entangled atrial and ventricular lead

An ultrasound study revealed a left ventricular ejection fraction (LVEF) of 35% with globally reduced wall motion and moderate tricuspid regurgitation. The atrial lead was not visible, and the shock coil was positioned at the lateral tricuspid annulus. Interrogation of ICD showed 122 inappropriate shocks due to sensing artifacts and exit-block in both leads. No ventricular or atrial arrhythmias were detected. After reprogramming the ICD to monitoring-only mode (ODO), the patient underwent an X-ray study, which confirmed the dislodgement of both leads (Fig. 1) and significant entanglement of the leads in the pocket area (Fig. 2). The patient was subsequently scheduled for revision due to Twiddler’s Syndrome.

### Past medical history

At the age of 57 years old, suffering from chronic renal insufficiency, he underwent an echocardiographic investigation, where a dilated cardiomyopathy (DCM) with reduced LVEF (35%) was diagnosed. A coronary angiography showed no coronary vessel disease. The patient was initiated on optimal medical therapy (OMT) for heart failure (HF) and chronic kidney disease (CKD). Despite an adequate medical therapy, impaired LVEF persisted and a dual chamber dual-coil ICD implantation was performed.

### Investigations

Laboratory results indicated slightly elevated retention values and N-terminal pro b-type Natriuretic Peptide (NT-pro-BNP) levels. However, these findings were consistent with those observed three months earlier.

### Procedural details with Heart Team involvement

**Fig. 3:**
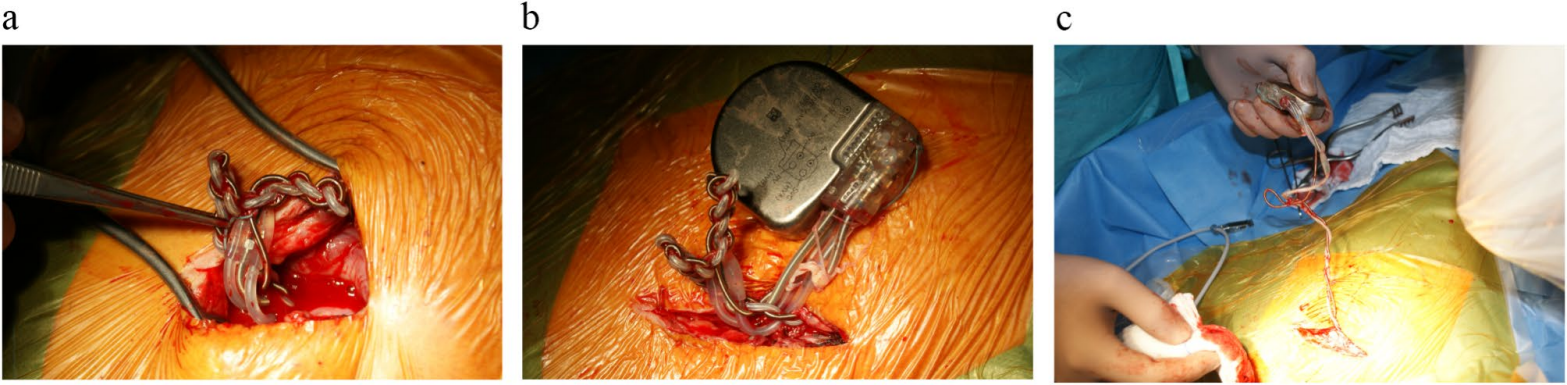
**A**, preparation of the “lead entanglement”. **B**, preparation of the ICD-generator. The suture attached to the CIED hardware is particularly striking. **C**, careful preparation and disentanglement of the ICD-leads before the extraction procedure could be conducted

Upon incision in the area of the generator placement, the entangled atrial and ventricular leads were exposed (Fig. 3A). The generator was mobilised from its fibrous capsule in the submuscular position. The suture used to secure the generator was found attached to the hardware (Fig. 3B). Following the mobilisation of the generator and the disentanglement of the leads, all fibrotic tissues attached to the leads were removed (Fig. 3C).

**Fig. 4:**
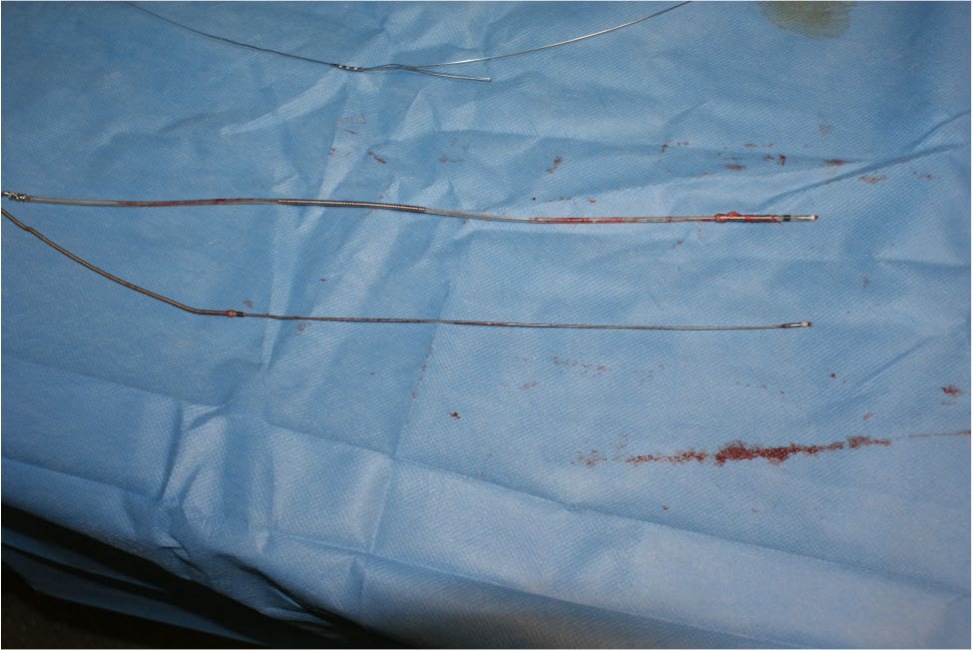
The successful and complete extraction of the ventricular dual-coil shock lead and the atrial lead

With the collaboration of a multidisciplinary heart team, including one cardiologist, one cardiac surgeon, one anaesthesiologist and one electrophysiologist, we proceeded with the extraction of the leads (Fig. 4) in conscious sedation and local anaesthesia. This was accomplished using a stylet and lead locking device (Spectranetics, Colorado Springs, Colorado, USA) and continuous irrigation with a taurolidine-containing antimicrobial solution (TauroPace, Tauropharm, Bavaria, Germany).

**Fig. 5:**
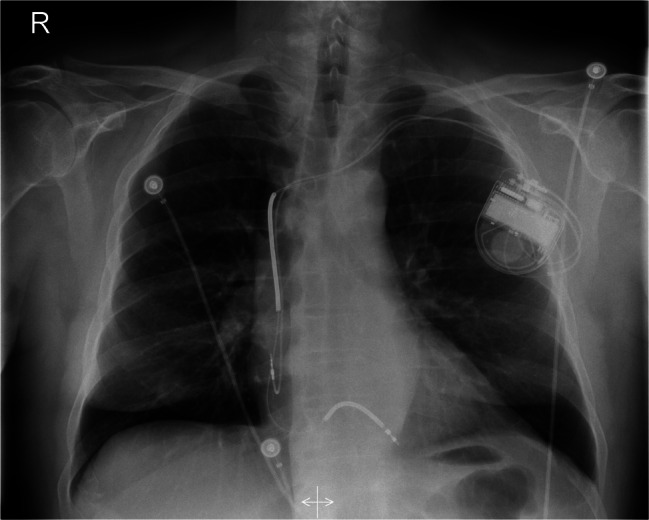
X-ray: placement of a DDD-ICD-system in the same session, with a new ventricular lead in the right apex and a new atrial lead in the right atrial appendage

After the hardware removal, a complete capsulectomy was performed, excising all fibrotic tissue encasing the old generator and leads. New leads and a new generator were then implanted (Fig. 5), again utilising the taurolidine-containing antimicrobial solution as an adjunct throughout the procedure. The use of taurolidine offered advantages over other antimicrobials, such as its broad-spectrum efficacy and reduced risk of resistance, which are crucial in preventing postoperative infections.

## Discussion and conclusions

DCM is a primary myocardial disease of non-ischemic origin (absence of obstructive coronary artery calcification). It is characterised by left ventricular dilatation and impaired left ventricular function. A non-ischemic origin accounts for up to 50% of patients with systolic heart failure (HF) in large HF studies, predominantly due to DCM. Despite advancements in HF treatment, excess mortality in DCM patients remains largely driven by LVEF deterioration and sudden cardiac death (SCD) due to arrhythmia. In the early 2000s the SCD-HeFT study [1] proved that prophylactic ICD implantation, on top of optimal medical therapy, reduces the overall mortality in patients with heart failure with reduced ejection fraction, compared to antiarrhythmic drugs and regardless of the underlying cardiomyopathy. Although this benefit has been clearly demonstrated in case of advanced left ventricular disfunction after myocardial infarction [2], in DCM the DANISH trial [3] downplayed the positive impact of this procedure and the related guidelines recommendations.

CIED placement itself potentially comes with a risk of complications or adverse events [4]. In various investigations, both terms are utilised interchangeably. A reported complication rate of 5–10% is contingent upon factors such as surveillance methods, definitional criteria, and the mode of reporting. Adverse events or complications can be categorised into those associated with the procedure itself and those related to the device. These may encompass pain at the site of insertion, pneumothorax, hematoma formation, vascular or valvular injury, venous thromboembolism, or venous obstruction, cardiac perforation, pericardial effusion, cardiac tamponade, lead dislodgement, generator displacement (Twiddler’s Syndrome), device malfunction, inappropriate shocks, diaphragmatic stimulation, superficial wound infection (minor CIED infection), major CIED infection (pocket infection and infective endocarditis), and mortality. Moreover, complications requiring invasive interventions, such as the insertion of a chest tube for pneumothorax and the early performance of revisions to access the generator (pocket), correspond with increasing procedural volumes. Revision of the CIED pocket is a delicate procedure as it substantially heightens the risk of CIED-related infections. Complications involving lead extraction necessitate specialised instruments and highly skilled personnel, contingent upon the duration the lead has been in place. Such resources may not be readily accessible in all geographical regions, leading to deviations from established guidelines during revisions, which, depending on the circumstances, can result in increased mortality rates [5]. 

Twiddler’s Syndrome is a rare complication, characterised by dislocation of the generator. The pathophysiological mechanisms underlying Twiddler’s Syndrome involve patient manipulation leading to inadvertent movement or rotation of the implanted device within its pocket. This manipulation can result from various factors such as body habitus, physical activities, or psychiatric conditions predisposing patients to repetitive twisting or manipulation of the device.

Twiddler’s Syndrome often leads to physical displacement of the leads and generator within the subcutaneous pocket. As the leads become twisted or dislodged, their positioning relative to cardiac structures may alter, resulting in abnormal sensing or pacing thresholds. This can lead to inappropriate sensing of signals, such as muscle or electrical artifacts, triggering the device to deliver inappropriate shocks. Furthermore, the displacement of leads may cause alterations in pacing impedance or capture thresholds, potentially leading to pacing failures or inadequate therapy delivery.

There is limited understanding concerning the pathogenesis associated with various other factors, including the types of suture materials used for securing the generator to the muscle fascia, the technique employed for suturing in the subdermal layer (interrupted versus continuous), as well as the materials of the generator and leads. Moreover, colonisation frequencies and their influence on the formation of a fibrous capsule around the generator, with or without calcification, remain poorly elucidated.

Initial placement in a submuscular location [6] or the utilisation of various devices aimed at rapid tissue integration of inert surfaces within the human body, such as an extracellular matrix envelope or an antibiotic-eluting mesh envelope, are strategies employed to address this issue. Nevertheless, outcomes have been inconsistent [7, 8]. 

In any revision procedure concerning the CIED generator and leads, whether involving lead extraction or replacement to adjust device functionality, complete removal of fibrotic tissues (capsulectomy) is advocated. This process aims to establish a fresh environment for the generator and associated hardware [9–12]. The approach varies for generator replacement necessitated by battery depletion. In these cases, accessing the fibrous capsule encompassing the old generator should entail subcutaneous preparation directed towards the capsule, commencing from a more cranial incision of the skin. Incisions spanning the generator pocket or extending in lateral, medial, or caudal directions in relation to the pocket should be avoided [13]. If the generator displaces or if there is a loss of subcutaneous tissue or fat, it may protrude through the skin, typically occurring in regions where skin integrity is compromised, such as scar tissue areas [11]. An incision at the caudal end of the fibrous capsule (i.e., break the pocket) during generator replacement for introducing immunocompetent cells is presently discouraged owing to the risk of hematoma formation [5, 14]. 

In the absence of any cardiac implantable electronic device (CIED) infection, a single-session extraction-reimplantation procedure may be utilised, particularly preferred for pacemaker-dependent patients. In instances of localised CIED infection, such as pocket infection, guidelines currently lack clear recommendations regarding the optimal timing for subsequent de novo placement [5, 14]. 

In the absence of persistent systemic infection (e.g., negative blood culture screenings or negative FDG-PET computed tomography along the transvenous leads), re-implantation of a new CIED in a contralateral position can be performed. This approach includes utilising infection prevention measures such as antibiotic-eluting mesh envelopes and taurolidine-containing antimicrobial compounds. Empiric antibiotic administration should be initiated based on local resistance patterns, followed by antibiotic administration tailored to susceptibility screening results [15]. 

Typically, prevailing guidelines encompass the following recommendations:

### Prevention

Patient education on avoiding device manipulation is crucial. Proper counselling about device care and potential consequences of manipulation can prevent Twiddler’s Syndrome.

### Regular follow-up

Guidelines advise regular visits to monitor device function and detect early signs of Twiddler’s Syndrome. Close surveillance helps identify lead dislodgement or device migration.

### Device positioning

Ensuring correct device placement minimises Twiddler’s Syndrome risk. Techniques like submuscular placement or additional anchoring sutures secure the device, reducing manipulation risk.

### Prompt intervention

Swift action is vital when Twiddler’s Syndrome is suspected. Interventions may include device repositioning (employing preventative measures against infection), lead extraction, or complete device replacement, based on syndrome severity.

### Multidisciplinary Approach

Collaboration involving the “Heart Team” is crucial for comprehensive management. A multidisciplinary approach tailors treatment to each patient’s needs.

### Follow-up

Recovery from the procedure was uneventful. During the follow-up, there were no clinical, image or laboratory findings suggesting any adverse event in relation to the procedure or the CIED.

### Conclusions

In times of an exponential increase of placement rates of complex CIED systems, complication rates have become a clinically relevant problem. Major complications after CIED placement increase morbidity, re-hospitalisation and mortality [4]. 

The Twiddler`s syndrome might lead to lead dislodgement and device malfunction. In our case this ultimately led to numerous inadequate shocks.

In absence of an infection, a single session extraction-re-implantation was feasible, employing a transvenous approach for lead extraction with specific tools and constantly disinfecting the surgical site and CIED hardware with an antimicrobial compound adjunct [16]. 

## Learning objectives

Gain insight into the aetiology, risk factors, and pathophysiology of Twiddler’s Syndrome.

Learn to recognise the clinical presentation and diagnostic criteria of Twiddler’s Syndrome, including signs of device dislodgement or migration.

Explore various management options for Twiddler’s Syndrome, including conservative measures, device repositioning, and surgical interventions like lead extraction or device replacement.

Understand preventive strategies to minimise the risk of Twiddler’s Syndrome, such as proper patient education on device care and counselling regarding potential consequences of device manipulation.

Appreciate the importance of a multidisciplinary approach involving cardiologists, cardiac electrophysiologists, cardiac surgeons, and other healthcare providers in the comprehensive management of Twiddler’s Syndrome.

Learn about the significance of regular follow-up visits and close surveillance in monitoring device function and detecting early signs of Twiddler’s Syndrome or associated complications.

Develop skills in effectively communicating with patients about Twiddler’s Syndrome, including educating them on risk factors, warning signs, and adherence to device care instructions.

## Data Availability

All data generated or analysed during this study are included in this published article.

## Abbreviations

CIED: Cardiac implantable electronic device
DCM: Dilated cardiomyopathy
ECG: Electrocardiogram
FDG-PET: Fluorodeoxyglucose positron emission tomography
HF: Heart failure
ICD: Implantable cardioverter defibrillator
LVEF: Left ventricular ejection fraction
n-ICMP: Non-ischemic cardiomyopathy
OMT: Optimal medical treatment
